# Neural stimulation suppresses mTORC1-mediated protein synthesis in skeletal muscle

**DOI:** 10.1126/sciadv.adt4955

**Published:** 2025-04-02

**Authors:** Ana G. Dumitras, Giorgia Piccoli, Frederik Tellkamp, Lena Keufgens, Martina Baraldo, Sabrina Zorzato, Laura Cussonneau, Leonardo Nogara, Marcus Krüger, Bert Blaauw

**Affiliations:** ^1^ Venetian Institute of Molecular Medicine (VIMM), Via Orus 2, 35129 Padova, Italy.; ^2^ Department of Biomedical Sciences, University of Padova, 35137 Padova, Italy.; ^3^ Institute for Genetics, Cologne Excellence Cluster for Aging and Aging-Associated Diseases (CECAD), University of Cologne, 50931 Cologne, Germany.; ^4^ Department of Pharmaceutical Sciences, University of Padova, 35137 Padova, Italy.

## Abstract

Skeletal muscle fibers are classified as glycolytic or oxidative, with differing susceptibilities to muscle wasting. However, the intracellular signaling pathways regulating fiber-specific muscle trophism remain unclear because of a lack of experimental models measuring protein synthesis. We developed a mouse model overexpressing a mutated transfer RNA synthetase in muscle fibers, enabling specific protein labeling using an artificial methionine substitute, which can be revealed through click chemistry. This model revealed that denervation increases protein labeling in oxidative muscle fibers through mammalian target of rapamycin complex 1 (mTORC1) activation, while deleting the mTORC1 scaffold protein Raptor reduces labeling in glycolytic fibers. On the other hand, increased muscle activity acutely decreases protein synthesis, accompanied by reduced mTORC1 signaling, glycogen depletion, and adenosine 5′-monophosphate kinase activation. Our findings identify nerve activity as an inhibitory signal for mTORC1-dependent protein synthesis in skeletal muscle, enhancing the understanding of fiber-specific responses to exercise and pathological conditions.

## Teaser

A murine model monitoring muscle protein synthesis shows that mTORC1 is inhibited by nerve activity and fiber type specificity.

## INTRODUCTION

It is well known that skeletal muscle is a very plastic tissue, responding rapidly to changes in activity levels, mechanical loading, or hormone levels (*1*). A major factor in determining functional properties of skeletal muscles is the activation pattern of motor neurons that recruit specific muscle fibers. The critical role played by neural input on muscle characteristics was already clear from cross-reinnervation experiments performed over 60 years ago (*2*). In these experiments, the slow motor nerve was connected to a fast muscle and vice versa, leading to a rapid conversion of contractile kinetics following the activity patterns imposed on the muscle. Mimicking the slow activity patterns (low stimulation frequency, with very high duty cycle) through electrical stimulation not only leads to a shift from fast-to-slow contractile characteristics, increases in mitochondrial content, and altered metabolic properties but, over time, also reduces muscle size (*3*, *4*). This link between smaller fiber size and high contractile activity, as observed in most slow, mitochondria-rich fibers, is also clearly visible in histological sections of mouse skeletal muscle. However, why highly active postural fibers are smaller and how this correlates with contractile activity are not clear.

One of the main signaling pathways regulating increases in muscle size and contractile properties is the insulin-like growth factor 1 (IGF1)-Akt-mammalian target of rapamycin complex 1 (mTORC1) signaling cascade (*5*). Increased signaling through IGF1 or increased activation of Akt is sufficient for a rapid and sustained increase in protein synthesis and hypertrophy in adult fast and slow skeletal muscle (*6*, *7*). Similarly, hyperactivation of mTORC1, by ablation of its inhibitor tuberous sclerosis complex 1 (TSC1) or by overexpression of Rheb (*8*), also leads to increased muscle size, even though this is only true for slow muscles in TSC1 knockout (ko) animals (*9*). Conversely, inhibiting mTORC1 signaling through rapamycin treatment or genetic deletion of the mTORC1 component Raptor does not induce rapid muscle atrophy in mice (*10*, *11*). Prolonged reduction of mTORC1 signaling, however, leads to the appearance of denervated and atrophic fibers, which is particularly evident in type 2B fibers (*12*), suggesting a functional link between fiber innervation and mTORC1 signaling.

Despite this connection between motor nerve activity, fiber size, and mTORC1 signaling, how nerve activity affects mTORC1 and subsequently regulates protein synthesis is currently not known. An important reason for this lack of knowledge is the fact that no good tools are available to monitor protein synthesis in vivo in a fiber type–specific manner. Isotope labeling techniques, like stable isotope labeling by amino acids in cell culture or radioactively labeled C14, can give important information but do not allow for a localization of the labeled proteins (*13*–*15*). Other commonly used approaches, like the incorporation of the antibiotic puromycin, is toxic and can only be used for the analysis of protein synthesis rates during short time windows (*8*).

In this study, we introduce a transgenic mouse model designed to monitor protein synthesis in vivo while manipulating muscle mTORC1 activation levels. This model uses skeletal muscle–specific overexpression of a mutated methionyl tRNA synthetase (MetRS), enabling the incorporation of the synthetic methionine analog azidonorleucine (ANL) in place of endogenous methionine. ANL-labeled proteins can be localized, quantified, and identified using click chemistry techniques (*16*). Using muscle denervation as a model for loss of activity, we discovered that muscle activity inhibits mTORC1-dependent protein synthesis. Intriguingly, we found that acute increases in activity levels through electrical stimulation inhibit protein synthesis. This inhibition is closely associated with reduced mTORC1 activity and decreased glycogen levels, suggesting a metabolic competition between contractile activity and protein synthesis. These findings provide insights into the complex relationship between muscle activity, mTORC1 signaling, and protein synthesis, highlighting potential metabolic trade-offs in skeletal muscle function. Together, our results show a causal link between contractile activity and its inhibitory effect on skeletal muscle anabolism through the regulation of mTORC1.

## RESULTS

### The muscle-specific MetRS mouse allows for labeling of newly synthesized muscle proteins

**Fig. 1.**
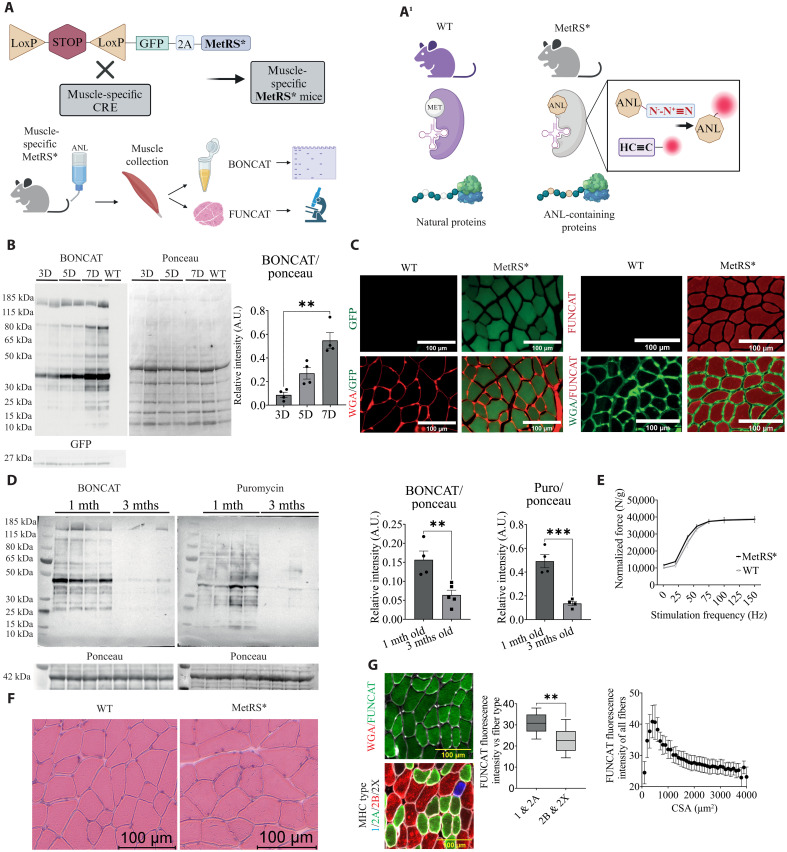
Muscle-specific MetRS mice allow for efficient labeling of newly synthesized proteins. (**A**) Scheme showing the generation of muscle-specific MetRS mice and workflow for identification and visualization of newly synthesized proteins. (**A**^**1**^) Schematic representation of the difference between mutated MetRS enzyme and WT. (**B**) Time-dependent increase in protein labeling after ANL administration (*n* = 4, means ± SEM; Kruskal-Wallis with Dunn’s post hoc test, **P < 0.01). A.U., arbitrary units. (**C**) Muscle cryosections stained for GFP (left panels, GFP in green and WGA in red) and FUNCAT (right panels, FUNCAT in red and WGA in green) in 7-day labeled MetRS animals versus WT. (**D**) Labeling intensity of ANL-containing proteins (BONCAT, left blot) and puromycin incorporation (right blot) in 1-month-old animals compared to 3-month-old ones followed by their quantifications (*n* = 4, means ± SEM, unpaired *t* test with statistical significance **P < 0.01 and ***P < 0.001). (**E**) Force production measurement (MetRS* versus WT after electrical stimulation through the sciatic nerve). (**F**) Hematoxylin and eosin muscle histology of WT animals (left) versus ANL-labeled Cre-MetRS animals (right). Scale bars, 100 μm. (**G**) Representative images (on the left) of differences in basal protein labeling (top image, FUNCAT in green and WGA in gray) in different fiber types (bottom image, MHC type 1 in blue, MHC type 2A in green, MHC type 2B in red, MHC type 2X in black, and WGA in gray). Scale bars, 100 μm. Fiber labeling quantification (graph on the left) (averages of 66 type 1 fibers, 190 type 2A fibers, 280 type 2B fibers, and 50 type 2X fibers were manually measured randomly across the slice for each biological replicate) and labeling versus CSA analysis in MetRS animals labeled with ANL for 1 week. An average of 8000 fibers was measured automatically for each biological replicate (means ± SEM, *n* = 5, unpaired *t* test with statistical significance **P < 0.01).

Despite being one the most important cellular processes, the tools to study changes in protein synthesis in vivo are still expectedly limited. Here, we generated a muscle-specific transgenic mouse line in which we overexpress a mutated MetRS, which allows for the incorporation of the synthetic amino acid ANL, instead of endogenous methionine, in specific time windows. These labeled proteins can be identified and visualized using click chemistry (Fig. 1A). To determine labeling efficiency, we added 30 mM ANL to the drinking water and performed a Western blot for labeled proteins. As can be seen in Fig. 1B, we observe a progressive increase in protein labeling, leveling off after 1 week. Performing an immunohistochemistry (IHC) approach for labeled proteins [fluorescent noncanonical amino acid tagging (FUNCAT)], we found that green fluorescent protein (GFP)–positive fibers (those expressing MetRS) show very strong labeling when compared to the unlabeled cre-negative control muscle (Fig. 1C). To get an idea whether increased labeling with ANL follows changes in protein synthesis rates, we compared the incorporation of puromycin and ANL during postnatal growth (1-month-old mice) and in adult, 3-month-old mice, as shown previously (*17*). As expected, muscles showing increased puromycin incorporation also have increased protein labeling with ANL, clearly suggesting that ANL incorporation follows changes in protein synthesis rates (Fig. 1D).

As different protein labeling approaches are characterized by toxic effects (like puromycin), we evaluated muscle function after labeling muscle proteins for 3 weeks. This time period is sufficiently long for most of the long-lived proteins in muscle to have had substantial turnover (*15*). Our in vivo measurements of muscle force and basic histological analysis (Fig. 1, E and F) revealed no significant alterations, indicating that the newly labeled proteins are nontoxic and maintain proper functionality. These findings are consistent with observations reported in previous studies (*16*, *18*). As muscles are composed of fibers with different activity patterns and metabolic characteristics, we determined basal protein labeling after 1 week of ANL administration. As can be seen in Fig. 1G, there are small yet significant differences in basal protein labeling, with stronger labeling in mitochondria-rich type 1 and 2A fibers.

### Loss of activity leads to an increase in protein labeling in mitochondria-rich type 1 and 2A fibers

**Fig. 2.**
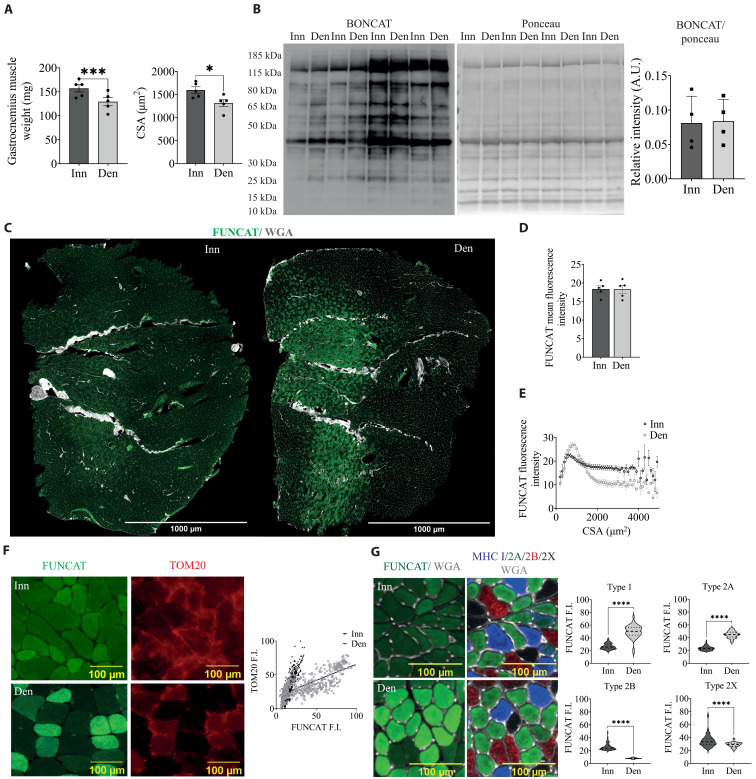
Denervation leads to an increase in protein labeling in type 1/2A fibers while decreasing in type 2B fibers. (**A**) Denervation (Den) leads to 15 to 20% loss in muscle mass (left) and fiber CSA (right). (**B**) Overall protein labeling (BONCAT left, ponceau right) by Western blotting of gastrocnemius muscle lysates of innervated or denervated limbs [means ± SEM, *n* = 5 (for muscle weight and CSA) and *n* = 4 (for Western blot quantification)]. For CSA measurements, each biological replicate represents the mean CSA of an average of 7000 fibers automatically measured (paired *t* test with statistical significance *P < 0.05 and ***P < 0.001) (**C**) FUNCAT images show an increase in labeling of specific fibers in denervated muscles. The innervated limb shown in the left panel is compared to the denervated one of the same mouse in the right panel (FUNCAT in green and WGA in gray). (**D**) Average fluorescence intensity of all fibers. An average of 7000 fibers was measured automatically for each biological replicate (means ± SEM, *n* = 3, paired *t* test with no statistical difference). (**E**) Quantification of labeling intensity based on fiber CSA in innervated and denervated limbs. (**F**) Increased labeling of fibers during denervation correlates with mitochondrial content. FUNCAT F.I. means fluorescence intensity. *n* = 4, simple linear regression with ****P < 0.0001. (**G**) Representative images (on the left) of labeling intensity (FUNCAT in green and WGA in gray) (MHC type 1 in blue, MHC type 2A in green, MHC type 2B in red, MHC type 2X in black, and WGA in gray, right panels). Scale bars, 100 μm. Quantification of FUNCAT intensity in different fiber types (on the right) (means ± SEM, *n* = 3). About 50 type 1 fibers, 50 type 2A fibers, 150 type 2B fibers, and 50 type 2X fibers were manually measured across the slice. Wilcoxon matched-pairs *t* test with statistical significance ****P < 0.0001.

To determine how protein labeling is affected by nerve activity, we performed unilateral denervation and added ANL to the drinking water for 1 week after sectioning of the nerve. As previously shown, we confirm that 1 week of denervation leads to a significant loss in muscle mass of about 15 to 20% (Fig. 2A). This loss in muscle size is not accompanied by a decrease in protein labeling in Western blotting (Fig. 2B), similar to what has been observed using other approaches of protein labeling (*13*, *14*). Considering the very significant differences in basal activity levels in different fiber types (*19*), we performed a FUNCAT analysis to see whether there were differences in labeling intensity between fiber types after denervation. Unexpectedly, we observed an increase in labeling in a subpopulation of fibers, while another group of fibers completely lost labeling (Fig. 2C). When analyzing in more detail the characteristics of these different fiber populations, we observed an increase in labeling intensity in smaller fibers as compared to bigger fibers (Fig. 2, D and E). Next, we performed costaining for FUNCAT and the mitochondrial protein TOM20. We found an increase in ANL incorporation in fibers with higher mitochondrial content (Fig. 2F), which is more pronounced in denervated muscles. Last, we performed an analysis on serial sections for fiber type and labeling intensity, performed on the same fibers. As can be seen in Fig. 2G, type 1 and 2A fibers showed a significant increase in protein labeling, while type 2B fibers lost signal following denervation (Fig. 2G).

### Increased protein labeling after denervation is mTORC1 dependent

**Fig. 3.**
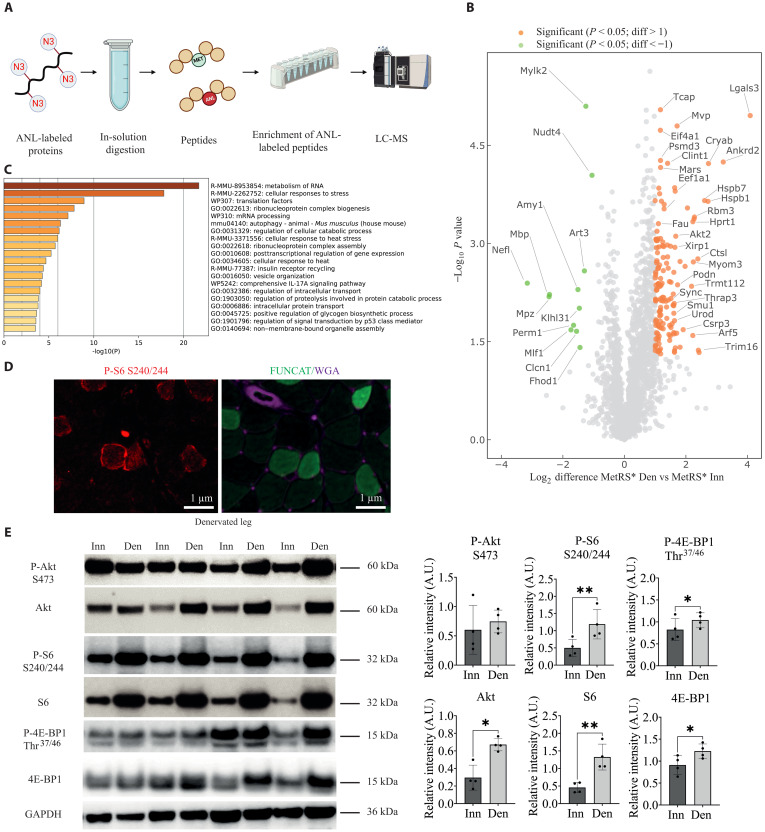
Denervation is accompanied by an increase in Akt-mTORC1 signaling and its downstream proteome. (**A**) BONCAT workflow used to identify ANL-labeled proteins in innervated and denervated muscles. LC-MS, liquid chromatography–mass spectrometry. (**B**) Volcano plot showing a significant amount of labeled proteins increase (orange) or decrease (green) comparing the innervated and denervated muscles from the same animals (*n* = 4 per group). Labeled proteins are at least twofold regulated and show *P* < 0.05. (**C**) Enrichment analysis of up-regulated labeled proteins 1 week after denervation shows a highly significant presence of processes previously shown to be regulated by activated mTORC1 signaling in skeletal muscle (*24*), like RNA metabolism and translation factors. (**D**) Representative image showing P-S6–positive fibers (left image, in red) that correspond to those fibers with higher ANL incorporation after denervation (right image, FUNCAT in green) in denervated limb. Scale bars, 1 μm. (**E**) Western blotting analysis of Akt-mTORC1 signaling in denervated contralateral muscles compared to control innervated contralateral muscles. For quantification of Western blots, *n* = 4 animals were used. Data are shown as the means ± SEM by paired *t* test with statistical significance *P < 0.05 and **P < 0.01.

Which pathway is responsible for this increased protein labeling in denervated mitochondria-rich fibers? To answer this question, we performed immunoprecipitation for labeled proteins followed by a mass spectrometry analysis (the workflow is depicted in Fig. 3A). The volcano plot in Fig. 3B, which indicates the fold change in labeled proteins comparing the innervated and denervated legs of the same animal, clearly shows many proteins that significantly increase (orange dots) after denervation. To identify affected pathways among these up-regulated proteins, we performed Gene Ontology enrichment analysis using the Metascape database (*20*). We identified RNA metabolism, autophagy, and translation as some of the top-ranked processes (Fig. 3C). These processes were linked to increased mTORC1 signaling in skeletal muscle in previous studies from our lab (*10*, *17*, *21*). Performing IHC staining for the phosphorylated form of ribosomal protein S6 (P-S6), a key downstream effector of mTORC1, we found that fibers with increased labeling after denervation also showed increased P-S6, suggestive of increased mTORC1 signaling in those fibers. Next, we performed a Western blotting analysis on innervated and denervated muscles and we confirmed an increase in signaling of the whole Akt-mTORC1 axis after denervation (Fig. 3, D and E), something already reported previously also by other groups (*22*, *23*).

**Fig. 4.**
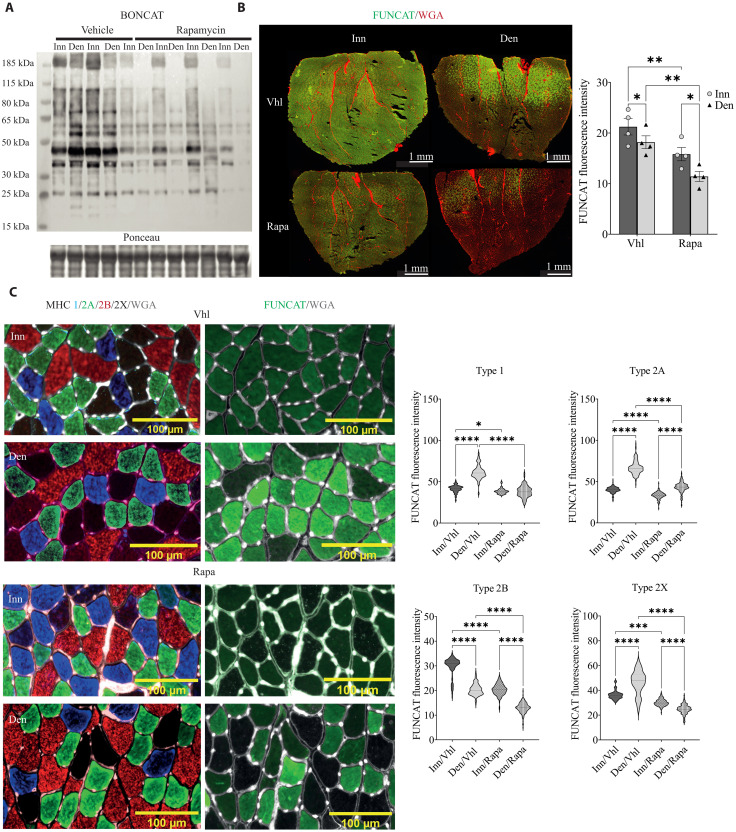
Increased labeling after denervation in type 1/2A fibers requires increased mTORC1 signaling. (**A**) Western blotting analysis for labeled proteins after 1 week of denervation in rapamycin- and vehicle-treated animals. Quantification in fig. S2B. (**B**) Representative images of FUNCAT (images on the left) in innervated or denervated muscles after rapamycin or vehicle treatment (FUNCAT in green and WGA in red). Quantification (graph on the right) of mean fluorescence intensity of single fibers (*n* = 4). An average of 5900 fibers was automatically measured for each biological replicate. Statistical analyses were performed using two-way ANOVA with Tukey’s post hoc test (*P < 0.05 and **P < 0.01). (**C**) Rapamycin treatment prevents the increase in protein labeling in type 1/2A fibers after denervation while decreasing even further the labeling in type 2B fibers. Representative images of serial IHC stained for MHC (MHC type 1 in blue, MHC type 2A in green, MHC type 2B in red, MHC type 2X in black, and WGA in gray, left panels) and FUNCAT (FUNCAT in green and WGA in gray, right panels). Around 50 type 1 fibers, 160 type 2A fibers, 160 type 2B fibers, and 50 type 2X fibers were manually measured across the slice. Quantification of FUNCAT in different fiber types (*n* = 4). Scale bars, 100 μm. Data are shown as the means ± SEM by two-way ANOVA with Tukey’s post hoc test (*P < 0.05, ***P < 0.001, and ****P < 0.0001).

Is increased mTORC1 signaling during denervation required for increased labeling in these postural, mitochondria-rich fibers? To address this issue, we repeated the denervation experiment in MetRS mice while treating them with either vehicle or rapamycin. Performing a Western blotting analysis for labeled proteins in rapamycin- and vehicle-treated animals, we found a significant reduction in the total amount of labeling in rapamycin-treated animals (Fig. 4A). While no differences were found between denervated and control muscles in the vehicle-treated animals, the rapamycin-treated group showed a significant reduction in labeled proteins in the denervated muscle compared to the contralateral one (fig. S2B). Next, we performed an IHC analysis for labeled proteins in both groups. As can be seen in Fig. 4B, there is a slight reduction in labeling intensity in innervated muscles when treated with vehicle or rapamycin, which is in line with previous observations revealing little effect of rapamycin administration on muscle mass and function during homeostatic conditions (*11*). The increase in protein labeling in mitochondria-rich fibers during denervation was completely abrogated by rapamycin treatment (Fig. 4C). A quantification of labeling intensity showed a partial or complete blunting of increased protein labeling in the denervated rapamycin-treated mice in type1/2A/2X fibers, while there is a significant decrease in type 2B fibers (Fig. 4, B and C). The reduction in basal protein labeling in the innervated muscles with rapamycin treatment is mainly observed in type 2B fibers, suggestive of higher basal mTORC1 signaling in these fibers. Together, these results clearly show that the increase in protein labeling after denervation in the more active, mitochondria-rich, muscle fibers is mTORC1 dependent.

### Nerve activity levels negatively affect mTORC1 activation

**Fig. 5.**
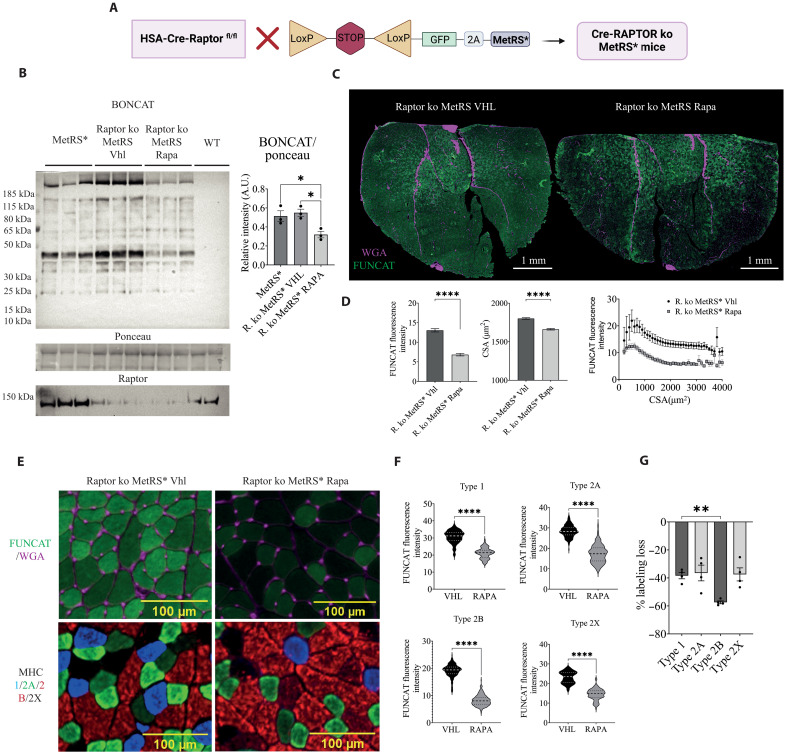
Acute, genetic loss of mTORC1 signaling in skeletal muscle reduces labeling predominantly in type 2B fibers. (**A**) Schematic representation of the generation of Raptor ko MetRS mice. (**B**) Western blot showing protein labeling in Raptor ko MetRS mice, treated with vehicle or with rapamycin compared to labeled MetRS animals (*n* = 5). Statistical analysis: ordinary one-way ANOVA with Tukey post hoc test (*P < 0.05). (**C**) FUNCAT representative images showing a general labeling decrease when comparing vehicle-treated Raptor ko MetRS animals (left panel) to rapamycin-treated animals (right panel) (FUNCAT in green and WGA in magenta; scale bars, 1000 μm). (**D**) Mean fluorescence intensity quantification of all fibers (graph on the left) and CSA versus FUNCAT analysis (graph on the right) show labeling reduction in rapamycin-treated animals accompanied by a mild atrophy (CSA analysis, graph in the middle). For this quantification, an average number of 6047 fibers were measured for each biological replicate. Statistical analyses were performed on *n* = 4 WT animals and *n* = 5 Cre-Raptor ko MetRS animals using two-tailed Student’s *t* test (****P < 0.0001). (**E**) Representative images of vehicle- or rapamycin-treated Raptor ko MetRS* animals: serial sections stained for FUNCAT (top panels, FUNCAT in green and WGA in magenta) and MYH isoforms (bottom panels, MHC type 1 in blue, MHC type 2A in green, MHC type 2B in red, and MHC type 2X in black). Scale bars, 100 μm. (**F**) Fiber type–dependent fluorescence quantification showing a general decrease in labeling in all fiber types. Statistical analyses were performed on *n* = 4 WT animals and *n* = 5 Cre-Raptor ko MetRS animals using two-tailed Student’s *t* test (****P < 0.0001). (**G**) The relative loss in labeling compared to control animals is more pronounced in 2B fibers (graph on the right). Statistical analyses were performed on *n* = 4 WT animals and *n* = 5 Cre-Raptor ko MetRS animals using one-way ANOVA with Tukey’s post hoc test and statistical significance **P < 0.01.

Since our data suggest that nerve activity inhibits mTORC1 signaling, we expect that relatively inactive fibers would have higher basal mTORC1 signaling. This hypothesis is also supported by the fact that rapamycin treatment reduces protein labeling, mainly in type 2B fibers (Fig. 4C). To address this issue properly, we used a genetic approach to reduce mTORC1 levels while monitoring protein labeling in a fiber type–specific manner. We crossed HSACreERT2-Raptor ko mice, described previously (*10*), with MetRS mice to allow us to label proteins after inducible deletion of Raptor in adult skeletal muscle (Fig. 5A). As we have shown previously (*10*), deletion of Raptor after 1 month does not show any overt phenotype; however, treatment of these mice with rapamycin for a week leads to an acute and complete inhibition of residual mTORC1 signaling. In Raptor ko mice treated with rapamycin, overexpression of a constitutively active form of Akt is no longer able to induce any fiber growth (*24*). As can be seen in Fig. 5B, protein labeling in Raptor ko mice is not different compared to controls, while labeling in the rapamycin-treated Raptor ko group is strongly reduced. Next, we conducted a FUNCAT analysis to investigate the fiber-specific effects of protein labeling loss. While rapamycin-treated Raptor ko animals exhibited a general reduction in labeling and mild atrophy (Fig. 5, C and D), certain fibers showed more pronounced labeling loss (Fig. 5C). Fiber type analysis revealed that inactive type 2B fibers displayed less protein labeling compared to postural type 1/2A fibers (Fig. 5, F and G). These findings align with the notion that basal mTORC1 activity is higher in type 2B fibers because of reduced activity–dependent mTORC1 inhibition (Figs. 3 and 4). This loss of protein labeling, specifically in type 2B fibers, is also reflected in the appearance of an early denervation phenotype almost exclusively in type 2B fibers in mice lacking mTORC1 signaling in skeletal muscle (*12*).

**Fig. 6.**
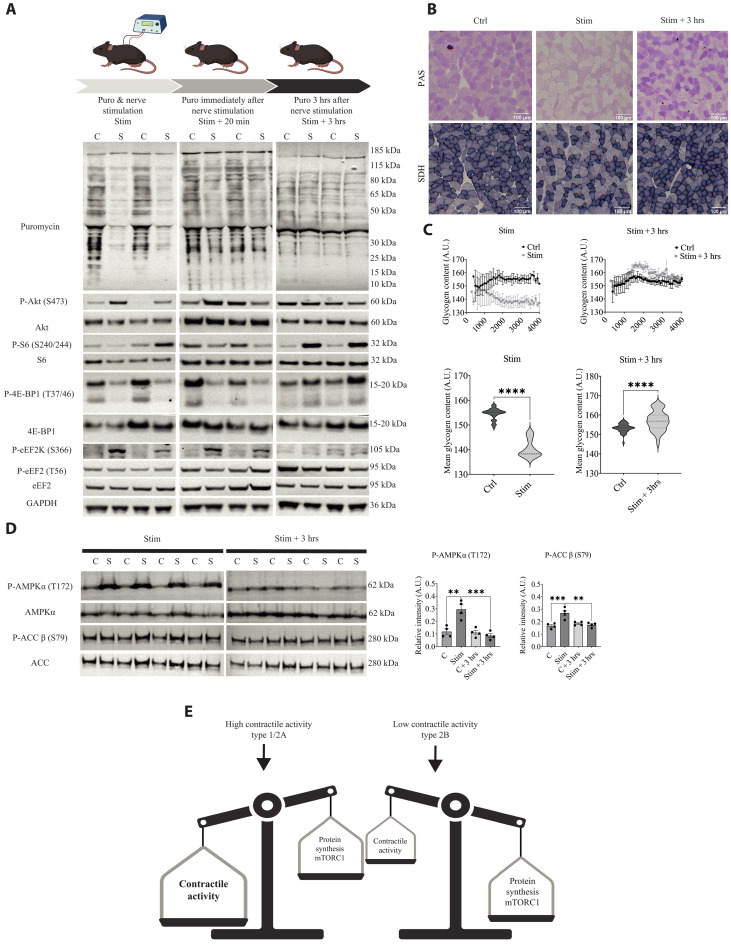
Electrical stimulation impairs mTORC1 signaling and protein synthesis. (**A**) Schematic representation of the electrical stimulation protocol used (upper part). Puromycin incorporation evaluated in three different time windows: the first overlapping with 20 min of stimulation (Stim), the second evaluated 20 min after the end of stimulation (Stim + 20 min), and the third evaluated 3 hours after the end of stimulation (Stim + 3 hrs). Representative image of puromycin incorporation immediately after stimulation (blot in the upper left image), 20 min after stimulation (Stim + 20 min, upper middle blot), and 3 hours after stimulation (Stim + 3 hrs, upper right blot). Representative images of blots for the Akt-mTORC1 pathway and elongation factors (lower part). Blot quantifications are in fig. S3A. (**B**) Representative images of serial stainings for glycogen content (PAS in upper panels) and SDH (lower panels) in control, stimulated muscles and muscles 3 hours after stimulation (Stim + 3 hrs). (**C**) Quantification of glycogen content versus fiber size (upper graphs) shows a clear reduction in glycogen content in bigger fibers after stimulation (upper graph on the left) compared to Stim +3 hrs (upper graph on the right). After stimulation, the average glycogen content is lower in stimulated fibers (lower graph on the left), while 3 hours after stimulation, the average glycogen content in stimulated fibers is higher than in the control muscle (lower graph on the right). Data are shown as the means ± SEM (*n* = 3, Wilcoxon matched-pairs *t* test, ****P < 0.0001). (**D**) Western blotting analysis for P-AMPK and its target P-ACC immediately after (Stim) and 3 hours after a bout of stimulation (Stim +3 hrs) (*n* = 4). Data are shown as the means ± SEM by two-way ANOVA with Tukey’s post hoc test (**P < 0.01 and ***P < 0.001). (**E**) Scheme depicting how contractile activity and mTORC1-dependent protein synthesis are two processes that are not occurring simultaneously, possibly due to competition for the same nutrient pool.

Given that reduced activity results in an mTORC1-dependent increase in protein labeling in postural muscle fibers, we hypothesized that elevating activity levels might produce the inverse effect—namely a reduction in mTORC1 activity and protein synthesis. To address this issue, we electrically stimulated the hindlimb of the leg for 20 min with a stimulation pattern similar to that of a slow motor neuron type (*19*). To determine protein synthesis rates in the stimulated and resting contralateral legs, we injected puromycin at the start of the stimulation protocol (Stim). As can be seen in Fig. 6 and fig. S3, we see a very marked decrease in protein synthesis rates in the stimulated leg compared to the control muscle, showing that activity inhibits protein synthesis rates. To determine the duration of protein synthesis suppression after the end of stimulation, we repeated the experiment; only this time, we injected puromycin at the end of the 20-min stimulation protocol (Stim + 20 min) or 3 hours later (Stim + 3 hrs). In both cases, muscles were harvested 20 min after the puromycin injection. While puromycin incorporation shows a trend toward an inhibition, already in 20 min following the single exercise bout, this difference is no longer significant (fig. S3A). At 3 hours after the stimulation bout, this trend is completely lost, and no differences are observed in protein synthesis rates in stimulated and control muscles.

Next, we performed an analysis of the signaling changes, focusing on Akt-mTORC1 signaling. Despite a strong increase in phosphorylation of Akt in samples taken immediately after the end of stimulation, downstream mTORC1 signaling was strongly impaired, as evidenced very clearly by the reduced phosphorylation of eukaryotic translation initiation factor 4E–binding protein 1 (4E-BP1) (Fig. 6A). In addition to translation initiation, also translation elongation, as evidenced by an increased phosphorylation of eukaryotic elongation factor 2 kinase (eEF2K), is strongly impaired at this time point. The phosphorylation changes observed immediately after stimulation are completely reversed [P-4E-BP1 (phosphorylated form of 4E-BP1)] or absent [P-Akt (phosphorylated form of Akt) and P-eEF2K (phosphorylated form of eEF2K)] after 3 hours, in line with the restoration of protein synthesis rates at this time point. Also, a significant increase in P-S6 levels at this time point is suggestive of increased mTORC1 signaling. Analysis of the glycogen content of muscles taken out immediately after stimulation or at Stim + 3 hrs shows that the 20-min stimulation protocol completely depletes fast glycolytic fibers of glycogen, which leads to glycogen supercompensation at Stim + 3 hrs (Fig. 6, B and C), suggesting a metabolic regulation for the inhibition of protein synthesis. Support for this hypothesis comes also from a Western blotting analysis for P-AMPK (phosphorylated form of adenosine 5′-monophosphate kinase) and its target P-ACC (phosphorylated form of acetyl-CoA carboxylase), showing a strong increase in both stimulated muscles immediately after the stimulation protocol, which returns to normal at Stim + 3 hrs (Fig. 6D). Together, our results suggest that nerve activity leads to a depletion of energy reserves, which in turn shuts off anabolic signaling and subsequent protein synthesis (Fig. 6E).

## DISCUSSION

Skeletal muscle is an extremely heterogeneous tissue, with each fiber type having very different contractile and metabolic properties (*1*). In the past 10 years, using various -omics approaches (*15*, *25*–*29*), it has become clear that also at the molecular level, fast and slow muscle fibers show major differences in transcriptional regulation and protein content. Expectedly, also the response to certain pathological stimuli can be quite different in fast and slow fibers (*30*). Despite this, very little is known about the signaling pathways that regulate muscle size and function in a fiber type–specific manner. Here, we show how changes in activity levels affect different fiber types and how this affects protein synthesis and mTORC1, a major regulator of adult muscle size.

The fiber type specificity in the response to altered mTORC1 signaling was already evident from the characterization of certain transgenic mouse models (*9*). Even though mice lacking the mTORC1 inhibitor TSC1 in skeletal muscle show a hyperactivation of mTORC1, almost all muscles show muscle atrophy instead of hypertrophy. This loss of muscle mass is the opposite from what is found after overexpression of one of the upstream activators of mTORC1, i.e., IGF1 or Akt (*6*, *7*), despite the fact that its anabolic response is mediated by mTORC1 (*24*, *31*). A possible explanation can be that hyperactivation of mTORC1 leads to reduced Akt phosphorylation, which is a critical step in stimulating glucose uptake into muscle fibers (*32*). Something similar is perhaps observed after electrical stimulation, as we find a strong increase in Akt phosphorylation, possibly due to the reduced mTORC1-S6K1 activity (*10*, *33*). This would suggest that reduced nutrient availability or uptake inhibits mTORC1 activation and protein synthesis, independently from Akt activation. In line with this thought, the type 1 and 2A fibers in TSC1 ko mice, which have the most mitochondria and, therefore, the most efficient adenosine 5′-triphosphate (ATP) production, show the least atrophy. The soleus muscle in these TSC1 ko mice is the only muscle showing hypertrophy, which is again in line with the fiber type composition of the soleus (type 2A and type 1), suggesting a metabolic brake on protein synthesis and muscle growth if nutrient/ATP availability is limited.

The competition between energy production and consumption, and how activity and protein synthesis are competing for the same energy reserves, is also suggested from our observed changes in protein synthesis after denervation. The highly active type 1/2A fibers have more mitochondria than the inactive type 2B fibers (type 2X fibers are almost always mixed fibers) and produce therefore more efficiently ATP to sustain their higher contractile activity. Once these fibers are no longer contracting, they no longer consume energy through cross-bridge cycling, possibly leading to increased energy availability to support processes like protein synthesis. This interpretation is in line with results observed in cardiac myocytes. A recent study showed that in cardiac myocytes, the level of ATP changes from one contraction to the next. These alterations are due to the energetic cost of the ion pumps involved in the maintenance of the membrane potential and excitation-contraction coupling, while the highest cost involves actin-myosin cross-bridge cycling (*34*). These results suggest that contractile activity consumes a significant quantity of ATP, therefore possibly limiting other costly cellular processes. It has been shown that protein synthesis is the intracellular process, which is most tightly linked to ATP supply, much more than ion cycling (*35*), supporting a view of metabolic competition between protein synthesis and muscle contraction. The coupling of the sarcoplasmic reticulum (SR) to the mitochondria appears critical, as loss of mitofusin 2 decreases ATP fluctuations between cardiomyocyte contractions. A link between SR-mitochondria coupling and ATP production was also observed recently in SEPN1-related myopathy, where the increased SR-mitochondria distance leads to reduced ATP production and subsequent protein synthesis (*36*). Restoration of ATP production and protein synthesis rescued both calcium handling and contractile properties in the highly active diaphragm muscle, suggesting an important link between energy supply and muscle function.

While mTORC1 signaling is clearly relevant for exercise-induced muscle growth/remodeling, there are multiple studies suggesting that there might be an inhibition of mTORC1 activity immediately after exercise (*37*). This inhibition occurred independently from the type of exercise that was performed. This is possibly due to the reduced availability of glucose immediately after mild exercise (*38*). Cells lacking TSC1/2, therefore having a constitutive activation of mTORC1, are particularly sensitive to glucose deprivation, as this reduces ATP levels, leading to increased cell death (*39*). Inhibition of mTORC1-dependent protein anabolism is sufficient to restore ATP levels and prevent excessive cell death in these cells. The reduction we observed in mTORC1 activity after electrical stimulation is more evident for 4E-BP1 than for RPS6. In the past, we have shown that 4E-BP1 and S6K1 have different roles in stimulating protein synthesis downstream of mTORC1 in skeletal muscle (*21**,* *24*), therefore suggesting an inhibition of specific cellular processes. One interesting hypothesis is linked to the metabolic rewiring after exercise. It has recently been shown that downstream of mTORC1, particularly 4E-BP1 is very important in regulating a metabolic switch from glucose metabolism to utilization of fatty acids during glucose starvation (*40*). 4E-BP1 inhibits the translation of ACC1 to reduce fatty acid synthesis and maintain redox homeostasis (*41*) during nutrient stress. While ACC1 is not the most expressed isoform in skeletal muscle, it has been suggested that after exercise, it can also play a role in exercise-induced muscle adaptations (*42*). In our stimulated muscles, it is likely that there is a shift toward fatty acid utilization, as glycogen depletion is highly significant. Three hours after the end of the stimulation, when glycogen stores are replenished, the inhibition of 4E-BP1–dependent translation is removed, and protein synthesis is restored. It is important to point out though that while glycogen depletion and AMPK activation closely follow the changes in mTORC1 signaling and protein synthesis, it has been shown that modulation of AMPK activity does not affect mTORC1 activation after electrical stimulation (*43*).

In addition to translation initiation, also translational elongation is inhibited by acute exercise, as evidenced by the very strong phosphorylation of eEF2K immediately afterward. Acute nutrient deprivation leads to a very robust activation of eEF2K and the subsequent inhibition of translational elongation, an important issue for tumor progression (*44*). It was also reported that eEF2K phosphorylation increases rapidly after electrical stimulation, and this closely follows reductions in protein synthesis in mice and humans (*45*, *46*). Furthermore, pharmacological inhibition of eEF2K ex vivo suggests that part of the reduced protein synthesis after increased muscle activity is through translational elongation. Together, these results suggest that acute nutrient depletion with increased muscle activity leads to a reduced protein synthesis, both by inhibiting translation initiation and elongation.

In conclusion, our study uses a series of innovative transgenic mouse models to demonstrate that acute contractile activity inhibits mTORC1-mediated protein synthesis. This inhibition results in significant variations in the basal activity levels across different muscle fiber types. Another major result from this study is the generation of a mouse model that can efficiently label muscle-specific proteins during specific time windows. Going forward, this innovative model will allow us to examine whether also more modest alterations in tonic activity, like muscle unloading, can affect mTORC1 activation and protein synthesis rates. The currently presented results not only show an important link between mTORC1 and muscle activity/exercise but also pave the way for the identification of fiber type–specific signaling pathways, a critical aspect for numerous neuromuscular diseases (*30*).

## MATERIALS AND METHODS

### Animals

For the present study, inducible muscle-specific GFP-MetRS L274G (mutant MetRS; MetRS*) (*16*) was generated by crossing MetRS* animals with mice expressing CreERT2 recombinase under the control of human skeletal actin promoter (HSA) (Fig. 1A). C57BL/6-Gt (ROSA)26Sor<tm1(CAG-GFP-Mars*L274G)Esm>/J animals were purchased from the Jackson Laboratory, Sacramento, CA. Genotyping was performed by polymerase chain reaction upon the Jackson Laboratory’s instructions (for the primer list and expected bands of MetRS mice, see fig. S1A). Upon tamoxifen administration, Cre recombinase fused to a mutated estrogen receptor leads to LoxP recombination and mutated methionyl-tRNA synthetase expression. Daily intraperitoneal tamoxifen injections were performed on 3-month-old animals for 1 week (3 mg/100 μl solubilized in ethanol/sunflower oil). HSA/CreERT2-Raptor ko MetRS animals were obtained by crossing MetRS animals with Raptor ko mice generated, as described previously (*10*). For MetRS* line characterization, both Cre-MetRS or wild-type MetRS (WT-MetRS) littermates were injected either with tamoxifen or vehicle. Three weeks after the end of tamoxifen treatment, ANL administration and proteome labeling were performed in vivo for 1 week. For muscle denervation, animals were anesthetized, and a 2-mm segment of the left limb sciatic nerve was excised. The nondenervated contralateral limb was used as a control. The animal study was reviewed and approved by the animal care committee (code number 225/202-PR).

### In vivo treatment protocols

Protein synthesis was measured using the SUnSET technique (*21*). Specifically, puromycin (0.04 μmol/g) was intraperitoneally injected 20 min before muscle collection. Dissected muscles were snap frozen simultaneously and kept at −80°C until processing. In the unilateral stimulation experiments, at the first time point, puromycin was injected at the start of the 20-min stimulation protocol. At the second one, puromycin was injected immediately after the last train of stimulation and tissues were taken out after 20 min. The third and last time point was obtained by injecting puromycin 3 hours after the last train of stimulation.

ANL H-L-Lys(N3)-OH*HCI (30 mM; Iris Biotech, HAA1625) was solubilized in tap water along with 0.7% maltose. The resulting solution was adjusted to pH 7 and sterilized on a filter with 0.22-μm pores. Water intake was measured daily (fig. S1B). To exclude ANL toxicity, mouse weight, lean mass, and fat mass were monitored using an EcoMRI machine (fig. S1C). Rapamycin treatment was performed by intraperitoneal injections at 2 mg/kg body weight every day for 1 week.

### In vivo muscle stimulation and force measurements

For electrical stimulation, C57BL/6J mice were deeply anesthetized with an intraperitoneal injection of ketamine (100 mg/kg) and xylazine (10 mg/kg) and, a few minutes later, an intramuscular injection of Altadol (5 to 10 mg/kg) as a pain relief treatment. The sciatic nerve of one hindlimb was exposed, and Teflon-coated multistranded steel wires were implanted on either side of the nerve. Electrostimulation was performed at 20-Hz frequency for 10 s every 30 s for a total duration of 20 min. Subsequent biochemical and histological analyses were performed on the gastrocnemius muscle.

Gastrocnemius muscle force was measured in living mice as previously described (*8*). Briefly, animals were anesthetized, and muscle contractile performance was measured in vivo using a 305B muscle lever system (Aurora Scientific Inc.). The measured absolute force was normalized to the gastrocnemius mass as an estimation of specific force. Animals were then euthanized by cervical dislocation, and muscles were collected, weighted, and frozen.

### Antibodies and Western blotting

The following antibodies were used for Western blotting: pAKT (Cell Signaling, S473; ref. 4060), AKT (Cell Signaling; ref. 9272), pS6 (Cell Signaling, S240/244; ref. 5364), S6 (Cell Signaling; ref. 2217), p4E-BP1 (Thr^37/46^) (Cell Signaling; ref. 2855), 4E-BP1 (Cell Signaling; ref. 9644), Phospho-eEF2 (Thr^56^) (Cell Signaling, 2331S), eEF2 Antibody (Cell Signaling, 2332S), Phospho-eEF2k (Ser^366^) (Cell Signaling; ref. 3691), eEF2k (Cell Signaling; ref. 3692), phospho-AMPKα (Thr^172^) (Cell Signaling; ref. 2535), anti-AMPKα (Cell Signaling; ref: 2532S), anti-Phospho-ACC (S79) (Cell Signaling; ref. 3661), anti-ACC (Cell Signaling; ref. 3676), GAPDH (glyceraldehyde-3-phosphate dehydrogenase; Abcam; ref. 8245), GFP (Invitrogen, A11122), anti-Puromycin (Invitrogen, MABE 343), and streptavidin-horseradish peroxidase (HRP; Invitrogen, SA10001). Tissues were homogenized in lysis buffer containing 50 mM tris, 150 mM NaCl, 10 mM MgCl_2_, 0.5 mM dithiothreitol, 1 mM EDTA, and 10% (w/v) glycerol supplemented with 1% (w/v) Triton, 1% (w/v) SDS, protease inhibitor (PI; 1:500 dilution of protease inhibitor cOmplete EDTA free, Roche), and phosphatase inhibitor (1:100 dilution of phosphatase inhibitor cocktail, Roche). Lysates were cleared by centrifugation and stored at −80°C until use.

Biorthogonal noncanonical amino acid tagging (BONCAT) was performed by treating 20 μg of proteins with 75 μM Acetylene-PEG_4_-Biotin (Jena Bioscience), 5.6 mM CuSO_4,_ 70 mM sodium ascorbate, 1 mM tris(3-hydroxypropyltriazolylmethyl)amine (THPTA; Sigma-Aldrich; ref. 762342). Biotinylated proteins were then separated by electrophoresis and immunoblotted on nitrocellulose. The resulting blot was blocked in 5% nonfat dry milk and incubated overnight with HRP-conjugated streptavidin (1:10000) or anti-GFP antibody (1:2000) in a solution of 5% nonfat dry milk. Chemiluminescence was detected using an Immobilon Classico or Crescendo western HRP substrate. Images were acquired using the ImageQuant LAS system.

### IHC and histology

Gastrocnemius cryosections (10 μm) were fixed in 4% (w/v) paraformaldehyde for 10 min and then permeabilized in 0.1% (w/v) Triton X-100 for 3 min. TOM20 (ref. SC-11–415) dilution at 1:50 in 1% goat serum (ref. SC-11–415) or p-S6 (S240/244; ref. 5364) dilution at 1:800 in 4% goat serum/phosphate-buffered saline (PBS) and 0.5% bovine serum albumin was followed by anti-rabbit secondary antibody incubation for 1 hour at 37°C in PBS. The fluorescent CuAAC reaction (FUNCAT) was performed overnight at 4°C using 1 mM THPTA (Sigma-Aldrich; ref. 762342), 7 mM CuSO_4_, 100 mM tris (pH 8), 100 mM sodium ascorbate, and 10 μM alkyne probe (AF488-Alkyne, Jena Bioscience; ref. CLK-1297). The following day, slices were washed in PBS (three times for 5 min) and further washed in PBS-EDTA-Tween [0.5 mM EDTA and 0.05% (w/v) Tween]. 4′,6-Diamidino-2-phenylindole was added to visualize nuclei, and Alexa Fluor 647–conjugated wheat germ agglutinin (WGA) that binds to sialic acid and *N*-acetylglucosaminyl residues was used to visualize cell membranes. Images were acquired using a Leica DM6B Upright LED Microscope equipped with a DFC 7000T camera. TOM20 quantification was performed by measuring the fluorescence intensity of TOM20 and click staining on the same muscle slice using ImageJ software.

For myosin heavy chain (MHC) isoform staining, fresh sections were blocked in mouse-on-mouse blocking reagent (Vector Laboratories, MKB-2213) following the manufacturer’s instructions. Primary antibodies BA-D5 (MyHC-slow), SC-71 (MyHC-2A), and BF-F3 (MyHC-2B) were purchased from Developmental Studies Hybridoma Bank and incubated overnight at 4°C in 1% bovine serum albumin and 4% goat serum in PBS. Secondary antibodies (Jackson ImmunoResearch, AffiniPure Mouse IgG Fab Fragments) were incubated in 4% goat serum in PBS for 1 hour at 37°C. CSA analyses were performed with SMASH (a MATLAB application) using a WGA staining for myofiber segmentation and myofiber contouring. For fiber type labeling quantifications, serial sections were stained for either FUNCAT or myosin isoforms. Using Fiji ImageJ software, same fibers were identified on two serial slices and the fluorescence intensity of about 500 fibers (averages of 50 type 1 fibers, 200 type 2A fibers, 200 type 2B fibers, and 50 type 2X fibers) was chosen randomly across different parts of the muscle and manually measured across the section. For total FUNCAT quantification and cross-sectional area (CSA) analysis, an average number of 6500 fibers were analyzed for each biological replicate.

Histology was performed on 10-μm–thick cryosections: For hematoxylin and eosin staining, a hematoxylin solution according to Mayer (Sigma-Aldrich, S1275) and eosin purchased from Leica were used; succinate dehydrogenase (SDH) staining was obtained by incubating slices for 30 min in nitrotetrazolium blue chloride solution (N6876-500MG). For periodic acid-Schiff (PAS) staining, slices were incubated in Carnoy fixative for 10 min, abundantly washed, and then incubated in a 0.5% periodic acid solution followed by a 10-min Schiff reagent incubation. PAS and SDH images were analyzed using Fiji ImageJ. Around 600 single fibers, chosen randomly around the section, were measured manually on sections taken from three different animals.

### Proteomics sample preparation

For proteomic analysis, frozen gastrocnemius muscle or tibialis anterior muscle was used and proteins were extracted with extraction buffer [0.1% SDS, 1% Triton X-100, 50 mM tris (pH 7.5), 150 mM NaCl, and 1 mM EDTA]. The protein concentration was determined using the Pierce 660-nm protein determination kit (ref. 22662), and 20 μg was subjected to acetone precipitation and afterward resuspended in digestion buffer [6 M urea, 2 M thiourea, and 10 mM Hepes (pH 8)]. For digestion, proteins were reduced with 10 mM dithiothreitol, alkylated with 55 mM chloroacetamide, and digested with LysC (Serva; ref. 20987.01) in a 1:50 enzyme-to-substrate ratio for 3 hours at room temperature. Subsequently, samples were diluted with 100 mM tris (pH 7.5) to a final urea + thiourea concentration of 2 M and digested with trypsin in a 1:100 enzyme-to-substrate ratio overnight. Samples were then desalted and stored on SDB-RPS double-layer (Affinisep; ref. SPE-Disks-Bio-RPS-M-47.20) stage tips until measurement.

For pull-down experiments, 200 μg was subjected to acetone precipitation and afterward resuspended in 1% SDS in PBS. Click reaction was performed using Biotin-PEG4-alkyne at a final concentration of 0.05 mM in the presence of 0.3 mM THPTA, CuSO_4_ (8.5 mg/ml), and sodium ascorbate (1.5 mg/ml) for 1 hour at room temperature while shaking. After the click reaction, samples were precipitated with acetone to remove excess reagents. Proteins were resuspended in extraction buffer. For precipitation, 15 μl of MyOne Streptavidin Dynabeads T1 (Invitrogen; ref. 65602) per sample was washed once in ultrapure water and then added to the samples. Samples were incubated overnight at room temperature with end-over-end rotation. Afterward, samples were washed five times with 200 μl of 6 M urea/thiourea in 10 mM Hepes, reduced with 5 mM tris(2-carboxyethyl)phosphine, alkylated with 40 mM chloroacetic acid, and digested with 0.5 μg of LysC for 3 hours. Furthermore, the samples were diluted with 50 mM ATP-binding cassette to 2 M urea/thiourea and digested with 0.5 μg of trypsin overnight. Digestion was stopped with 1% formic acid, and peptides were loaded on SDB-RPS double-layer stage tips until measurement.

### Proteomics sample measurements and analysis

Samples were eluted with 2% ammonia solution in 60% acetonitrile into 96-well plates and dried in a vacuum concentrator. Afterward, samples were resuspended in 10 μl of 5% formic acid and 2% acetonitrile. Two microliters was injected for each measurement. Samples were measured on a Thermo Orbitrap Q Exactive Plus instrument coupled to a Thermo Easy nLC 1200 HPLC instrument. Each sample was measured with a 90-min reverse-phase gradient in a data-independent acquisition mode (DIA). Resulting raw files from pull-down experiments were converted to mzML files using the MSconvertGUI tool from ProteoWizard. Spectral matching was performed with DIA-NN 1.8.1 (*47*) using a UniProt database containing mouse canonical proteins and default settings plus “heuristic protein inference”, “--report-lib-info” for further data analysis, and implicit protein grouping by protein names. The DIA-NN output report was analyzed and rearranged by in-house built Rscript using MaxLFQ for protein quantification. Further analysis was done using Perseus version 16.2.3 (*48*) and InstantClue version 0.11.2 (*49*).

### Statistical analysis

In the denervation and stimulation experiments, the contralateral leg was used as a control. The experiments were performed with at least four animals per condition. We used different cohorts for analysis, and for each experimental cohort, both males and females were considered.

Statistical analyses of these data were performed using GraphPad Prism 9.5.0 (GraphPad, La Jolla, CA) followed by paired or unpaired two-tailed *t* test or one-way analysis of variance (ANOVA) test. *P* values are reported in the figures, and *P* < 0.05 was considered statistically significant.

Proteomics data were analyzed in Perseus using Welch’s *t* test with permutation-based false discovery rate control to account for multiple hypothesis testing. We used an *S*_0_ value of 0.1 for statistical testing and considered false discovery rates below 5% as significant.

## Supplementary Materials

### This PDF file includes:

Figs. S1 to S3 Table S1 References
