# Supplementary material for: Neural stimulation suppresses mTORC1-mediated protein synthesis in skeletal muscle
Source: Sci Adv. 2025 Apr 2;11(14):eadt4955. doi: 10.1126/sciadv.adt4955 (PMC11963989; doi:10.1126/sciadv.adt4955)
Supplement: Supplementary file 1 — Figs. S1 to S3 Table S1 References [file sciadv.adt4955_sm.pdf]

Science Advances
AAAS

Supplementary Materials for

Neural stimulation suppresses mTORC1-mediated protein synthesis in skeletal muscle

Ana G. Dumitras *et al.*

Corresponding author: Bert Blaauw, bert.blaauw@unipd.it; Marcus Krüger, marcus.krueger@uni-koeln.de

Sci. Adv. **11**, eadt4955 (2025)
DOI: 10.1126/sciadv.adt4955

This PDF file includes:

Figs. S1 to S3
Table S1
References

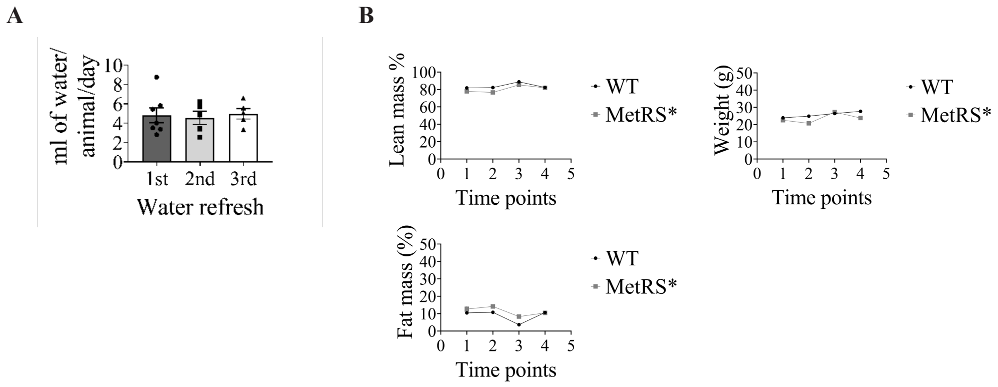

Fig. S1. ANL labelling does not show toxicity A) No differences observed in ANL-containing water intake. **B)** Weight, lean mass and fat mass (as determined by EchoMRI (50)) during a three-week treatment show no significant differences between MetRS and wildtype animals. Time points represent each a different measure over the three weeks treatment.

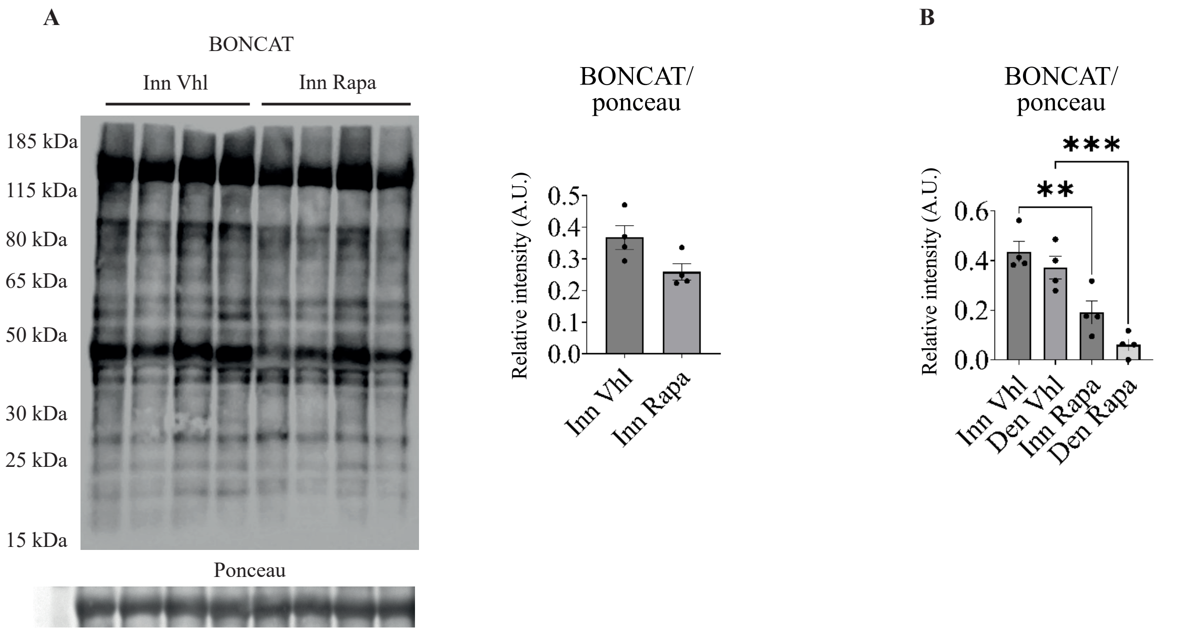

Fig S2. Rapamycin treatment reduces protein labelling in both innervated and denervated muscles A) Western blot showing ANL-labeled proteins in vehicle and rapamycin treated animals (n=4) , data expressed as mean ± SEM, statistical analysis performed: unpaired t-test with no statistical significance. **B)** BONCAT quantification of Fig 4A Western Blot (n=4). Data are shown as mean ± SEM. Statistical analyses were performed using one-way ANOVA with Tukey's post-hoc test with statistical significance: *P<0,05 **P<0.01).

A

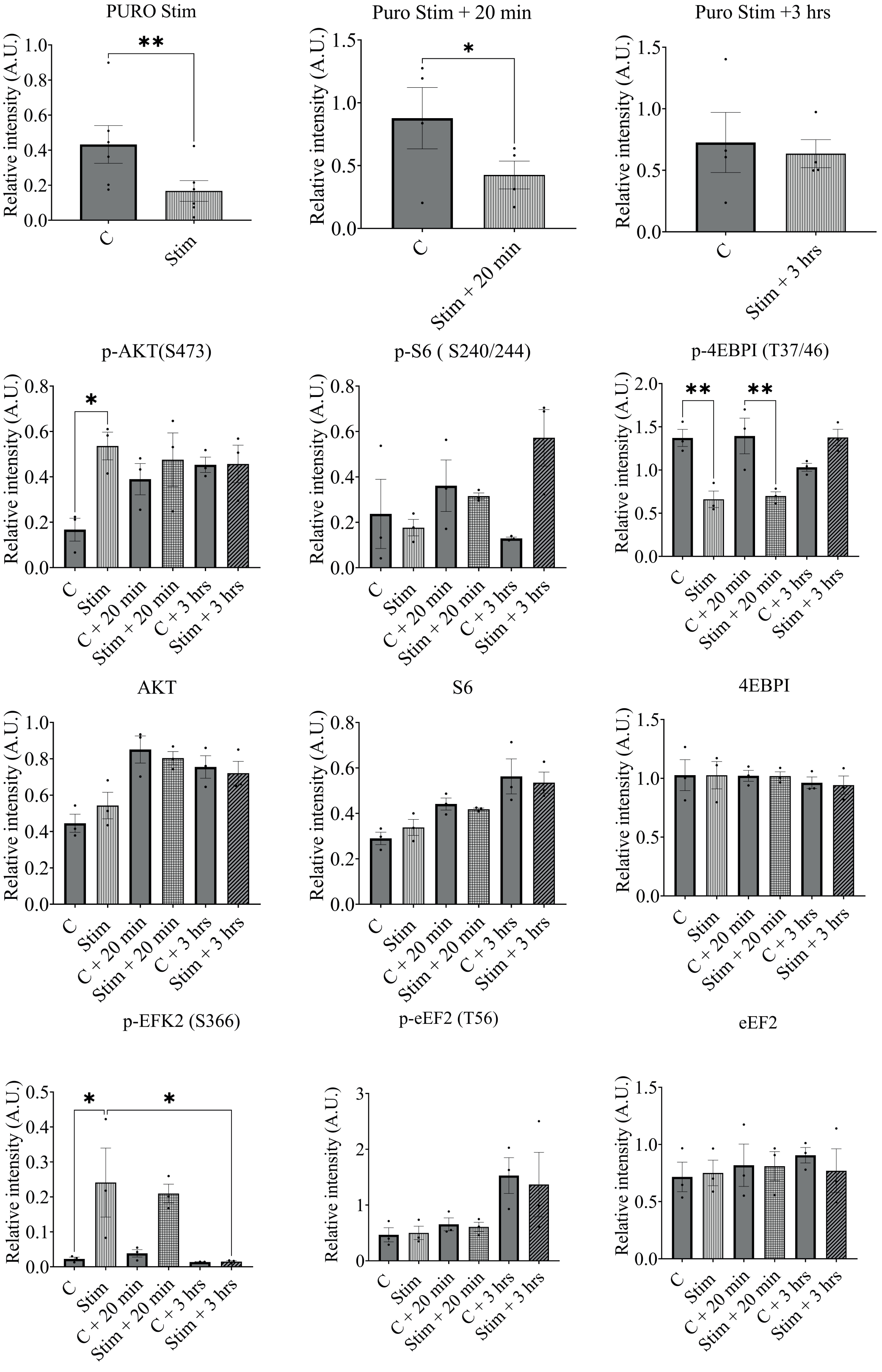

Fig. S3. Quantification changes in protein synthesis markers after electrical stimulation. A) Quantification of western blots form Fig 6A(n=3). Data are shown as mean ± SEM. Statistical analyses performed: paired t-test were performed for puromycin western blots and ordinary one-way ANOVA with Tukey-s post-hoc test for the remaining blots. Statistical significance: *P<0,05 **P<0.01.

Name	Jackson denomination	Primers
Mutant forward	12614	ACCACTACCAGCAGAACACC
Wild type	21306	CTGGCTTCTGAGGACCG
Mutant reverse	26209	GGCAGATTGCACTAGCAGAG
Wild type reverse	oIMR9021	CCGAAAATCTGTGGGAAGTC

Table S1.Primers used for MetRS* animals genotyping.

REFERENCES AND NOTES

1. B. Blaauw, S. Schiaffino, C. Reggiani, Mechanisms modulating skeletal muscle phenotype. *Compr. Physiol.* **3**, 1645–1687 (2013).

2. A. J. Buller, J. C. Eccles, R. M. Eccles, Interactions between motoneurones and muscles in respect of the characteristic speeds of their responses. *J. Physiol.* **150**, 417–439 (1960).

3. S. Salmons, G. Vrbova, The influence of activity on some contractile characteristics of mammalian fast and slow muscles. *J. Physiol.* **201**, 535–549 (1969).

4. R. H. Westgaard, T. Lomo, Control of contractile properties within adaptive ranges by patterns of impulse activity in the rat. *J. Neurosci.* **8**, 4415–4426 (1988).

5. S. Schiaffino, C. Reggiani, T. Akimoto, B. Blaauw, Molecular mechanisms of skeletal muscle hypertrophy. *J. Neuromuscul. Dis.* **8**, 169–183 (2021).

6. A. Musaro, K. McCullagh, A. Paul, L. Houghton, G. Dobrowolny, M. Molinaro, E. R. Barton, H. L. Sweeney, N. Rosenthal, Localized Igf-1 transgene expression sustains hypertrophy and regeneration in senescent skeletal muscle. *Nat. Genet.* **27**, 195–200 (2001).

7. B. Blaauw, M. Canato, L. Agatea, L. Toniolo, C. Mammucari, E. Masiero, R. Abraham, M. Sandri, S. Schiaffino, C. Reggiani, Inducible activation of Akt increases skeletal muscle mass and force without satellite cell activation. *FASEB J.* **23**, 3896–3905 (2009).

8. C. A. Goodman, D. M. Mabrey, J. W. Frey, M. H. Miu, E. K. Schmidt, P. Pierre, T. A. Hornberger, Novel insights into the regulation of skeletal muscle protein synthesis as revealed by a new nonradioactive in vivo technique. *FASEB J.* **25**, 1028–1039 (2011).

9. P. Castets, S. Lin, N. Rion, S. Di Fulvio, K. Romanino, M. Guridi, S. Frank, L. A. Tintignac, M. Sinnreich, M. A. Ruegg, Sustained activation of mTORC1 in skeletal muscle inhibits constitutive and starvation-induced autophagy and causes a severe, late-onset myopathy. *Cell Metab.* **17**, 731–744 (2013).

10. M. Baraldo, A. Geremia, M. Pirazzini, L. Nogara, F. Solagna, C. Turk, H. Nolte, V. Romanello, A. Megighian, S. Boncompagni, M. Kruger, M. Sandri, B. Blaauw, Skeletal muscle mTORC1 regulates neuromuscular junction stability. *J. Cachexia Sarcopenia Muscle* **11**, 208–225 (2020).

11. D. J. Ham, A. Borsch, K. Chojnowska, S. Lin, A. B. Leuchtmann, A. S. Ham, M. Thurkauf, J. Delezie, R. Furrer, D. Burri, M. Sinnreich, C. Handschin, L. A. Tintignac, M. Zavolan, N. Mittal, M. A. Ruegg, Distinct and additive effects of calorie restriction and rapamycin in aging skeletal muscle. *Nat. Commun.* **13**, 2025 (2022).

12. M. Baraldo, S. Zorzato, A. H. T. Dondjang, A. Geremia, L. Nogara, A. G. Dumitras, M. Canato, L. Marcucci, H. Nolte, B. Blaauw, Inducible deletion of raptor and mTOR from adult skeletal muscle impairs muscle contractility and relaxation. *J. Physiol.* **600**, 5055–5075 (2022).

13. K. A. Kobak, M. M. Lawrence, G. Pharaoh, A. K. Borowik, F. F. Peelor III, P. D. Shipman, T. M. Griffin, H. Van Remmen, B. F. Miller, Determining the contributions of protein synthesis and breakdown to muscle atrophy requires non-steady-state equations. *J. Cachexia Sarcopenia Muscle* **12**, 1764–1775 (2021).

14. H. M. Argadine, N. J. Hellyer, C. B. Mantilla, W.-Z. Zhan, G. C. Sieck, The effect of denervation on protein synthesis and degradation in adult rat diaphragm muscle. *J. Appl. Physiol.* **107**, 438–444 (2009).

15. F. Lang, S. Aravamudhan, H. Nolte, C. Turk, S. Holper, S. Muller, S. Gunther, B. Blaauw, T. Braun, M. Kruger, Dynamic changes in the skeletal muscle proteome during denervation-induced atrophy. *Dis. Model. Mech.* **10**, 881–896 (2017).

16. B. Alvarez-Castelao, C. T. Schanzenbacher, C. Hanus, C. Glock, S. Tom Dieck, A. R. Dorrbaum, I. Bartnik, B. Nassim-Assir, E. Ciirdaeva, A. Mueller, D. C. Dieterich, D. A. Tirrell, J. D. Langer, E. M. Schuman, Cell-type-specific metabolic labeling of nascent proteomes in vivo. *Nat. Biotechnol.* **35**, 1196–1201 (2017).

17. M. G. Pereira, K. A. Dyar, L. Nogara, F. Solagna, M. Marabita, M. Baraldo, F. Chemello, E. Germinario, V. Romanello, H. Nolte, B. Blaauw, Comparative analysis of muscle hypertrophy models reveals divergent gene transcription profiles and points to translational regulation of muscle growth through increased mTOR signaling. *Front. Physiol.* **8**, 968 (2017).

18. O. D. Shahar, E. M. Schuman, Large-scale cell-type-specific imaging of protein synthesis in a vertebrate brain. *eLife* **9**, e50564 (2020).

19. R. Hennig, T. Lomo, Firing patterns of motor units in normal rats. *Nature* **314**, 164–166 (1985).

20. Y. Zhou, B. Zhou, L. Pache, M. Chang, A. H. Khodabakhshi, O. Tanaseichuk, C. Benner, S. K. Chanda, Metascape provides a biologist-oriented resource for the analysis of systems-level datasets. *Nat. Commun.* **10**, 1523 (2019).

21. M. Marabita, M. Baraldo, F. Solagna, J. J. M. Ceelen, R. Sartori, H. Nolte, I. Nemazanyy, S. Pyronnet, M. Kruger, M. Pende, B. Blaauw, S6K1 is required for increasing skeletal muscle force during hypertrophy. *Cell Rep.* **17**, 501–513 (2016).

22. P. Castets, N. Rion, M. Theodore, D. Falcetta, S. Lin, M. Reischl, F. Wild, L. Guerard, C. Eickhorst, M. Brockhoff, M. Guridi, C. Ibebunjo, J. Cruz, M. Sinnreich, R. Rudolf, D. J. Glass, M. A. Ruegg, mTORC1 and PKB/Akt control the muscle response to denervation by regulating autophagy and HDAC4. *Nat. Commun.* **10**, 3187 (2019).

23. J.-S. You, K. Kim, N. D. Steinert, J. Chen, T. A. Hornberger, mTORC1 mediates fiber type-specific regulation of protein synthesis and muscle size during denervation. *Cell Death Discov.* **7**, 74 (2021).

24. M. Baraldo, L. Nogara, G. A. Dumitras, A. H. Tchampda Dondjang, A. Geremia, M. Scalabrin, C. Turk, F. Telkamp, L. Zentilin, M. Giacca, M. Kruger, B. Blaauw, Raptor is critical for increasing the mitochondrial proteome and skeletal muscle force during hypertrophy. *FASEB J.* **35**, e22031 (2021).

25. S. Kallabis, L. Abraham, S. Muller, V. Dzialas, C. Turk, J. L. Wiederstein, T. Bock, H. Nolte, L. Nogara, B. Blaauw, T. Braun, M. Kruger, High-throughput proteomics fiber typing (ProFiT) for comprehensive characterization of single skeletal muscle fibers. *Skelet Muscle* **10**, 7 (2020).

26. M. Murgia, N. Nagaraj, A. S. Deshmukh, M. Zeiler, P. Cancellara, I. Moretti, C. Reggiani, S. Schiaffino, M. Mann, Single muscle fiber proteomics reveals unexpected mitochondrial specialization. *EMBO Rep.* **16**, 387–395 (2015).

27. M. Dos Santos, S. Backer, B. Saintpierre, B. Izac, M. Andrieu, F. Letourneur, F. Relaix, A. Sotiropoulos, P. Maire, Single-nucleus RNA-seq and FISH identify coordinated transcriptional activity in mammalian myofibers. *Nat. Commun.* **11**, 5102 (2020).

28. M. Canepari, M. Maffei, E. Longa, M. Geeves, R. Bottinelli, Actomyosin kinetics of pure fast and slow rat myosin isoforms studied by in vitro motility assay approach. *Exp. Physiol.* **97**, 873–881 (2012).

29. M. Canepari, R. Rossi, O. Pansarasa, M. Maffei, R. Bottinelli, Actin sliding velocity on pure myosin isoforms from dystrophic mouse muscles. *Muscle Nerve* **40**, 249–256 (2009).

30. S. Ciciliot, A. C. Rossi, K. A. Dyar, B. Blaauw, S. Schiaffino, Muscle type and fiber type specificity in muscle wasting. *Int. J. Biochem. Cell Biol.* **45**, 2191–2199 (2013).

31. C. Rommel, S. C. Bodine, B. A. Clarke, R. Rossman, L. Nunez, T. N. Stitt, G. D. Yancopoulos, D. J. Glass, Mediation of IGF-1-induced skeletal myotube hypertrophy by PI(3)K/Akt/mTOR and PI(3)K/Akt/GSK3 pathways. *Nat. Cell Biol.* **3**, 1009–1013 (2001).

32. N. Jaiswal, M. G. Gavin, W. J. Quinn III, T. S. Luongo, R. G. Gelfer, J. A. Baur, P. M. Titchenell, The role of skeletal muscle Akt in the regulation of muscle mass and glucose homeostasis. *Mol. Metab.* **28**, 1–13 (2019).

33. S. H. Um, F. Frigerio, M. Watanabe, F. Picard, M. Joaquin, M. Sticker, S. Fumagalli, P. R. Allegrini, S. C. Kozma, J. Auwerx, G. Thomas, Absence of S6K1 protects against age- and diet-induced obesity while enhancing insulin sensitivity. *Nature* **431**, 200–205 (2004).

34. P. Rhana, C. Matsumoto, Z. Fong, A. D. Costa, S. G. Del Villar, R. E. Dixon, L. F. Santana, Fueling the heartbeat: Dynamic regulation of intracellular ATP during excitation-contraction coupling in ventricular myocytes. *Proc. Natl. Acad. Sci. U.S.A.* **121**, e2318535121 (2024).

35. F. Buttgereit, M. D. Brand, A hierarchy of ATP-consuming processes in mammalian cells. *Biochem. J.* **312**, 163–167 (1995).

36. S. Germani, A. T. Van Ho, A. Cherubini, E. Varone, A. Chernorudskiy, G. M. Renna, S. Fumagalli, M. Gobbi, J. Lucchetti, M. Bolis, L. Guarrera, I. Craparotta, G. Rastelli, G. Piccoli, C. de Napoli, L. Nogara, E. Poggio, M. Brini, A. Cattaneo, A. Bachi, T. Simmen, T. Cali, S. Quijano-Roy, S. Boncompagni, B. Blaauw, A. Ferreiro, E. Zito, SEPN1-related myopathy depends on the oxidoreductase ERO1A and is druggable with the chemical chaperone TUDCA. *Cell Rep. Med.* **5**, 101439 (2024).

37. R. Blazev, C. S. Carl, Y.-K. Ng, J. Molendijk, C. T. Voldstedlund, Y. Zhao, D. Xiao, A. J. Kueh, P. M. Miotto, V. R. Haynes, J. P. Hardee, J. D. Chung, J. W. McNamara, H. Qian, P. Gregorevic, J. S. Oakhill, M. J. Herold, T. E. Jensen, L. Lisowski, G. S. Lynch, G. T. Dodd, M. J. Watt, P. Yang, B. Kiens, E. A. Richter, B. L. Parker, Phosphoproteomics of three exercise modalities identifies canonical signaling and C18ORF25 as an AMPK substrate regulating skeletal muscle function. *Cell Metab.* **34**, 1561–1577.e9 (2022).

38. J. Axsom, T. TeSlaa, W. D. Lee, Q. Chu, A. Cowan, M. R. Bornstein, M. D. Neinast, C. R. Bartman, M. C. Blair, K. Li, C. Thorsheim, J. D. Rabinowitz, Z. Arany, Quantification of nutrient fluxes during acute exercise in mice. *Cell Metab.* **36**, 2560–2579.E5 (2024).

39. A. Y. Choo, S. G. Kim, M. G. Vander Heiden, S. J. Mahoney, H. Vu, S.-O. Yoon, L. C. Cantley, J. Blenis, Glucose addiction of *TSC* null cells is caused by failed mTORC1-dependent balancing of metabolic demand with supply. *Mol. Cell* **38**, 487–499 (2010).

40. T. Levy, K. Voeltzke, L. Hruby, K. Alasad, Z. Bas, M. Snaebjörnsson, R. Marciano, K. Scharov, M. Planque, K. Vriens, S. Christen, C. M. Funk, C. Hassiepen, A. Kahler, B. Heider, D. Picard, J. K. M. Lim, A. Stefanski, K. Bendrin, A. Vargas-Toscano, U. D. Kahlert, K. Stühler, M. Remke, M. Elkabets, T. G. P. Grünewald, A. S. Reichert, S.-M. Fendt, A. Schulze, G. Reifenberger, B. Rotblat, G. Leprivier, mTORC1 regulates cell survival under glucose

starvation through 4EBP1/2-mediated translational reprogramming of fatty acid metabolism. *Nat. Commun.* **15**, 4083 (2024).

41. S.-M. Jeon, N. S. Chandel, N. Hay, AMPK regulates NADPH homeostasis to promote tumour cell survival during energy stress. *Nature* **485**, 661–665 (2012).

42. H. M. O'Neill, J. S. Lally, S. Galic, T. Pulinilkunnil, R. J. Ford, J. R. B. Dyck, B. J. van Denderen, B. E. Kemp, G. R. Steinberg, Skeletal muscle ACC2 S212 phosphorylation is not required for the control of fatty acid oxidation during exercise. *Physiol. Rep.* **3**, e12444 (2015).

43. J. R. Knudsen, Z. Li, K. W. Persson, J. Li, C. Henriquez-Olguin, T. E. Jensen, Contraction-regulated mTORC1 and protein synthesis: Influence of AMPK and glycogen. *J. Physiol.* **598**, 2637–2649 (2020).

44. G. Leprivier, M. Remke, B. Rotblat, A. Dubuc, A.-R. F. Mateo, M. Kool, S. Agnihotri, A. El-Naggar, B. Yu, S. P. Somasekharan, B. Faubert, G. Bridon, C. E. Tognon, J. Mathers, R. Thomas, A. Li, A. Barokas, B. Kwok, M. Bowden, S. Smith, X. Wu, A. Korshunov, T. Hielscher, P. A. Northcott, J. D. Galpin, C. A. Ahern, Y. Wang, M. G. McCabe, V. P. Collins, R. G. Jones, M. Pollak, O. Delattre, M. E. Gleave, E. Jan, S. M. Pfister, C. G. Proud, W. B. Derry, M. D. Taylor, P. H. Sorensen, The eEF2 kinase confers resistance to nutrient deprivation by blocking translation elongation. *Cell* **153**, 1064–1079 (2013).

45. A. J. Rose, B. Bisiani, B. Vistisen, B. Kiens, E. A. Richter, Skeletal muscle eEF2 and 4EBP1 phosphorylation during endurance exercise is dependent on intensity and muscle fiber type. *Am. J. Physiol. Regul. Integr. Comp. Physiol.* **296**, R326–R333 (2009).

46. A. J. Rose, T. J. Alsted, T. E. Jensen, J. B. Kobbero, S. J. Maarbjerg, J. Jensen, E. A. Richter, A Ca^{2+}-calmodulin-eEF2K-eEF2 signalling cascade, but not AMPK, contributes to the suppression of skeletal muscle protein synthesis during contractions. *J. Physiol.* **587**, 1547–1563 (2009).

47. V. Demichev, C. B. Messner, S. I. Vernardis, K. S. Lilley, M. Ralser, DIA-NN: Neural networks and interference correction enable deep proteome coverage in high throughput. *Nat. Methods* **17**, 41–44 (2020).

48. S. Tyanova, T. Temu, P. Sinitcyn, A. Carlson, M. Y. Hein, T. Geiger, M. Mann, J. Cox, The Perseus computational platform for comprehensive analysis of (prote)omics data. *Nat. Methods* **13**, 731–740 (2016).

49. H. Nolte, T. D. MacVicar, F. Tellkamp, M. Kruger, Instant clue: A software suite for interactive data visualization and analysis. *Sci. Rep.* **8**, 12648 (2018).

50. Geremia A, Sartori R, Baraldo M, Nogara L, Balmaceda V, Dumitras GA, Ciciliot S, Scalabrin M, Nolte H, Blaauw B. Activation of Akt-mTORC1 signaling reverts cancer-dependent muscle wasting. *Journal of Cachexia, Sarcopenia and Muscle*, 2022 Feb;**13**(1):648-661
